# Helping the decision maker effectively promote various experts’ views into various optimal solutions to China’s institutional problem of health care provider selection through the organization of a pilot health care provider research system

**DOI:** 10.1186/1478-4505-11-11

**Published:** 2013-04-04

**Authors:** Liyang Tang

**Affiliations:** 1 Department of Economics, School of Economics and Management, Tsinghua University, Beijing 100084, China; 2 Graduate School of Business, Columbia University, New York, NY 10027, USA

**Keywords:** Analytic network process, China’s Health Care System Reform, Decision maker, Economy, Effectiveness, Efficiency, Equity, Experts’ views, Health care provider research system, Health care provider selection, Institutional problem

## Abstract

**Background:**

The main aim of China’s Health Care System Reform was to help the decision maker find the optimal solution to China’s institutional problem of health care provider selection. A pilot health care provider research system was recently organized in China’s health care system, and it could efficiently collect the data for determining the optimal solution to China’s institutional problem of health care provider selection from various experts, then the purpose of this study was to apply the optimal implementation methodology to help the decision maker effectively promote various experts’ views into various optimal solutions to this problem under the support of this pilot system.

**Methods:**

After the general framework of China’s institutional problem of health care provider selection was established, this study collaborated with the National Bureau of Statistics of China to commission a large-scale 2009 to 2010 national expert survey (n = 3,914) through the organization of a pilot health care provider research system for the first time in China, and the analytic network process (ANP) implementation methodology was adopted to analyze the dataset from this survey.

**Results:**

The market-oriented health care provider approach was the optimal solution to China’s institutional problem of health care provider selection from the doctors’ point of view; the traditional government’s regulation-oriented health care provider approach was the optimal solution to China’s institutional problem of health care provider selection from the pharmacists’ point of view, the hospital administrators’ point of view, and the point of view of health officials in health administration departments; the public private partnership (PPP) approach was the optimal solution to China’s institutional problem of health care provider selection from the nurses’ point of view, the point of view of officials in medical insurance agencies, and the health care researchers’ point of view.

**Conclusions:**

The data collected through a pilot health care provider research system in the 2009 to 2010 national expert survey could help the decision maker effectively promote various experts’ views into various optimal solutions to China’s institutional problem of health care provider selection.

## Background

The year 2007 could be taken as the start of China’s Health Care System Reform, as it was in this year that the Chinese government made an unprecedented commitment to improve the quality and expand the breadth of health care through a 2020 reform scheme. Through this reform scheme a new national health care system covering every Chinese citizen would be realized [1,2]. In 2009 this commitment was re-proposed and drew further attention. Driven by the Inter-Ministerial Health Care Reform Group of the State Council, the aggressive 2020 goal that was adjusted in 2009 sat atop the following four major pillars of the draft scheme [3-6]:

1. Improving public health;

2. Enhancing the medical insurance and welfare system;

3. Improving the quality and efficiency of hospitals through the enhanced management and monitoring;

4. Improving basic medical services.

The main aim of China’s Health Care System Reform was to help the decision maker find the optimal solution to China’s institutional problem of health care provider selection [7]. In the implementation of China’s Health Care System Reform, the expectation of the whole society forced various health care institutions to provide their products and services for the public more efficiently, more economically, more effectively, and more equally. The decision maker came to realize that the traditional government’s regulation-oriented health care provider approach may no longer be the optimal approach, making a reform for the selection of health care providers urgently needed [3-7]. In fact the selection of health care providers has also received considerable attention in the literature, and its increasing importance has forced the decision maker to rethink both the traditional evaluation strategies and the traditional regulation strategies on China’s institutional problem of health care provider selection [8-11]. On the basis of the current situation of China’s health care system, the possible alternative approaches include the public private partnership (PPP) approach and the market-oriented health care provider approach [7]. Obviously, each type of health care provider has its own difficulties and problems in achieving the exciting 2020 goal, but many optimal mechanisms and systems that can easily solve these difficulties and problems are also found in China’s health care system [3-7]. Since inefficiency, diseconomy, ineffectiveness, and inequity issues in China’s health care system must be taken seriously, efficiency, economy, effectiveness, and equity can be taken as the major sub-goals of China’s institutional problem of health care provider selection (there may be other sub-goals of China’s institutional problem of health care provider selection, but they are usually the relatively minor sub-goals of this problem) [3-7,12]. Since there are tradeoffs between these sub-goals with respect to each type of health care provider, the decision maker needs to balance the advantages and disadvantages of various optimal mechanisms and systems in order to find the optimal solution to China’s institutional problem of health care provider selection that takes all sub-goals as a whole [7,13].

Various experts have had various views on China’s institutional problem of health care provider selection, and their views have usually been collected through research projects, public-opinion solicitation of the government, and media interviews. Specifically, the early literature suggested two opposite solutions to China’s institutional problem of health care provider selection. One solution was putting health care providers under the strict and complete administration of the government, and its supporters included a small proportion of medical practitioners (doctors, pharmacists, nurses, and hospital administrators), a large proportion of officials (officials in health administration departments and medical insurance agencies), and a small proportion of health care researchers (in colleges, universities, and research departments). The other solution was the marketization of health care providers, and its supporters included a large proportion of medical practitioners, a small proportion of officials, and a large proportion of health care researchers [7,10,11,14].

The characteristics of putting health care providers under the strict and complete administration of the government were as follows: in financial terms, all health care institutions were managed in the form of “separation of revenue and expenditure” by the health administration departments; in medical device/medicine terms, the health administration departments implemented the uniform bidding and purchasing for all health care institutions; in personnel terms, the health administration departments were responsible for the appointment/hiring of both the deans and the deputy deans of all health care institutions; in price terms, the prices of health care services, medical devices, and medicines were under the unified control of the government’s price departments; generally speaking, in this administration system, a health care institution that was only a budget unit of the government did not have an independent legal person qualification [10,11,14]. The marketization of health care providers supported the following point of view: in any health care field, as long as the competition exists, the market must be established, and both public and private health care institutions must play fairly in the same market environment [9,15], because only when the government opens up the health care market and expedites the privatization of health care, it is possible to efficiently allocate a large number of medical resources [8]. Here the privatization of health care meant that private for-profit/non-profit health care institutions could enter the health care market freely, and various experts had the most controversial views on this sub-problem of China’s institutional problem of health care provider selection [14-18].

Based on the above two possible solutions, the Public Policy Research Department of China’s Economic Reform Research Society further put forward three comparative points of view, involving the administrative-oriented point of view, the conservative market-oriented point of view, and the progressive market-oriented point of view [7]. The administrative-oriented point of view that was mainly supported by officials suggested that the Chinese government should use the public power to limit the business scope of private health care institutions in order to make them as the complementary part of the health care market and maintain the dominant position of public health care institutions in the health care market [7]. The conservative market-oriented point of view that was mainly supported by health care researchers was in favor of an open market that the certified private health care institutions could enter, but it designated a framework of “strengthening the control of both the large-scale and small-scale private health care institutions, and loosening the control of the medium-scale private health care institutions” [7]. The progressive market-oriented point of view that was mainly supported by medical practitioners did not support the restriction of the scope of the health care privatization, and it believed that the business scopes of both public and private health care institutions should depend on the competition result of a level playing field, rather than the government’s administrative arrangement [7,19,20].

Up till now, an authoritative study that helped the decision maker effectively promote various experts’ views into various optimal solutions to China’s institutional problem of health care provider selection has been lacking. This situation is mainly due to the lack of an authoritative health care provider research system, since such a system can most efficiently collect the data for determining the optimal solution to China’s institutional problem of health care provider selection from various experts. This situation is also partly due to the fact that few implementation methodologies can meet the requirement of the difficult problem-solving process, since this problem-solving process involves an analysis of interdependent activities whose critical attributes include intangible elements. In the literature, a number of implementation methodologies have been applied to solve China’s institutional problem of health care provider selection, such as qualitative methods [7] and statistical methods [21]; however, none of them could handle the possible interactions among decision factors/constraints in the complex multi-stage setting in this problem, so they were not the effective implementation methodologies that could help the decision maker effectively promote various experts’ views into various optimal solutions to China’s institutional problem of health care provider selection.

The construction of the health care provider research system has been the weakness of the construction of China’s health care system for a long time and this problem has significantly blocked China’s Health Care System Reform, as the decision maker could not effectively promote various experts’ views into various optimal solutions to China’s institutional problem of health care provider selection without the organization of an authoritative health care provider research system [22-29]. In order to help the decision maker determine the optimal solution to China’s institutional problem of health care provider selection on the basis of various experts’ views on this problem, a pilot health care provider research system was recently organized in China’s health care system. Since this pilot health care provider research system could efficiently collect the data for determining the optimal solution to China’s institutional problem of health care provider selection from various experts, the purpose of this study was to apply the optimal implementation methodology to help the decision maker effectively promote various experts’ views into various optimal solutions to this problem under the support of this pilot system.

## Methods

### The general framework of China’s institutional problem of health care provider selection

In order to generally examine various health care provider approaches based on various experts’ views on China’s institutional problem of health care provider selection, the general framework of this problem was established, incorporating the following important considerations:

• Tangible/intangible attributes and strategic attributes;

• Dependencies between attributes;

• Preferences for attributes with respect to different types of health care;

• Preferences and inter-relationships of health care providers with respect to different stages of health care.

**Figure 1:**
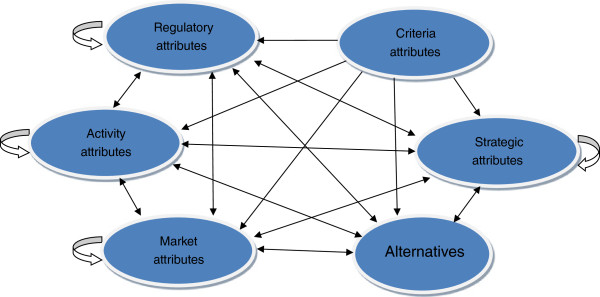
The general framework of China’s institutional problem of health care provider selection.

In Figure 1, a collection of similar attributes was referred to as a cluster, and there were six clusters below the overall goal on the basis of the above important considerations: the activity cluster, the regulatory cluster, the market cluster, the alternative cluster, the criterion cluster, and the strategic cluster. The dependency relationship between attributes within a cluster was called inner-dependency, denoted by a directed loop at the cluster. A two-way dependency relationship between attributes in two different clusters was called inter-dependency, denoted by a two-way directed arc between the clusters.

The following two aspects for the activity cluster, the regulatory cluster, and the market cluster should be taken seriously: complementarities of resources and capabilities, the commitment and mutuality of social/business needs. Complementarities of resources and capabilities helped the health care system gain new resources and capabilities via strategic alliances and strategic networks, resulting in access to new information, new technologies, and new markets [30,31]. The commitment and mutuality of social/business needs were the critical aspect that dictated how well resources and capabilities contributed to the sustainable partnership among participants of the health care market. These two aspects were also inter-related.

The following six subsections developed the underlying concepts that constituted the generic set of factors for the general framework of China’s institutional problem of health care provider selection.

#### Selection of activity attributes

Activity attributes reflect the specifications/characteristics embodied in health care related activities, and they are organized into four parts, namely, basic health care, non-basic health care, health care and insurance system, and medicine/medical device supply.

#### Selection of regulatory attributes

Regulatory attributes are organized into two parts, namely, management system, and supervision/monitoring.

In order to solve the institutional problem of health care provider selection, the decision maker must consider whether different health care providers should possess differentiated medical devices, differentiated human capitals, and differentiated medical technology advantages that are complementary to each other. In order to ensure a sustainable partnership among participants of the health care market, the decision maker must evaluate health care providers’ service reputation, organizational processes, abilities to offer customers adaptations/customizations, intra-organizational compatibilities, etc. [32-34]. In the regulatory cluster, the management system attribute mainly refers to the administrative control of the health care market, and the supervision/monitoring attribute mainly refers to the regulatory control of the health care market.

#### Selection of market attributes

Market attributes are organized into three parts, namely, compensation choice, health insurance system, and financing.

Here the compensation choice attribute mainly refers to the government’s choice between compensating the supply-side and compensating the demand-side in the health care market. The health insurance system, regulated by the government’s medical insurance agency and insurance regulatory commission, is a major part of the health care market, and it is responsible for the health care reimbursement of the public/the insured person. The financing attribute mainly refers to the structure of participants in health care financing.

#### Selection of alternatives

The alternative cluster is composed of alternative approaches for the selection of health care providers. Each alternative approach is assessed with respect to all attributes in the activity cluster, the regulatory cluster, and the market cluster [35]. On the basis of both various experts’ various views on China’s institutional problem of health care provider selection in the literature and the current situation of China’s health care system, the most viable alternative approaches include the traditional government’s regulation-oriented health care provider approach, the PPP approach, and the market-oriented health care provider approach. These alternative approaches, distinguished by all attributes in the activity cluster, the regulatory cluster, and the market cluster, are explored with respect to China’s institutional problem of health care provider selection:

Alternative approach 1: The traditional government’s regulation-oriented health care provider approach

1. Basic health care: it is under both the comprehensive executive order and the complete control of the government, only the public health care institution acts as the supplier of basic health care.

2. Non-basic health care: it is mainly administrative-oriented and partly market-oriented, under the control of the government the private for-profit/non-profit health care institution can enter the non-basic health care market, the government uses the public power to maintain the dominant position of the public health care institution and limit the business scope of the private health care institution to make it as the complementary part of the non-basic health care market.

3. Health care and insurance system: a publicly-funded health care system without the “internal market reform” is implemented, and it is responsible for both basic health care and public health of the whole society. The government implements a mandatory social health insurance system as the backbone of the medical security system, under the control of the government both the medical aid system and the commercial health insurance system play the role of complementary health insurance system.

4. Medicine/medical device supply: the desired medicine/medical device is mainly supplied by the public interest institution, and the incentives of the public interest institution for medicine/medical device supply are meeting its national/social objectives. Here other major indicators such as medicine/medical device production facilities, R&D, production management, and supply chain should also be assessed for this attribute.

5. Management system: the government imposes the complete command and control (involving the implementation of “separation of revenue and expenditure”) on the public health care institution, and the business scope of the public health care institution is limited to a certain extent by the government. The private for-profit/non-profit health care institution that is subject to the government’s regulation suffers discriminatory treatment in many aspects, since it is treated only as the complementary part of the health care market by the government.

6. Supervision/Monitoring: the setting of standards for both price and quality of health care and the work of addressing the potential market failures are mainly done by the government. The aim of the government’s supervision and monitoring is to achieve a guaranteed minimum outcome of health care provision.

7. Compensation choice: the government mainly compensates the supply-side in the health care market, and the public health care institution is compensated to provide appropriate health care for the public through a top-down performance assessment by the government.

8. Health insurance system: a mandatory social health insurance system is implemented as the backbone of the medical security system, and it is responsible for the reimbursement of basic health care of the public. Under the government’s control, both the commercial health insurance system and the medical aid system are only responsible for the reimbursement of non-basic health care of the insured person.

9. Financing: the financing of health care mainly refers to public financing from both the central/local government and the state owned enterprise.

Alternative approach 2: The PPP approach

1. Basic health care: the government, the public health care institution, and the private for-profit/non-profit health care institution jointly participate in defining both the social objectives and the implementation methods of basic health care. Here, the public health care institution acts as the major supplier, and the private for-profit/non-profit health care institution acts as the auxiliary supplier.

2. Non-basic health care: both the social objectives and the implementation methods of non-basic health care are also defined by the government, the public health care institution, and the private for-profit/non-profit health care institution, but in the non-basic health care market the role of each type of health care institution mainly depends on the competition result of a level playing field.

3. Health care and insurance system: a publicly-funded health care system without the “internal market reform” is currently implemented, and it is responsible for both basic health care and public health of the whole society, but the “internal market reform” will be implemented step by step, and ultimately the publicly-funded health care system will be replaced by the social health system. The government currently implements a mandatory social health insurance system as the backbone of the medical security system, but the mandatory social health insurance system will be gradually replaced by a non-mandatory multilevel public health insurance system. Both the medical aid system and the commercial health insurance system are treated as complementary health insurance systems by the government.

4. Medicine/medical device supply: the desired medicine/medical device is mainly supplied by both the public interest institution and the private for-profit/non-profit institution. The incentives of the public interest institution for medicine/medical device supply are meeting its national/social objectives, whereas those of the private for-profit institution are making profit, and those of the private non-profit institution are meeting its social/organizational objectives. Here, other major indicators such as medicine/medical device production facilities, R&D, production management, supply chain, and private medicine/medical device firms should also be assessed for this attribute.

5. Management system: the government imposes the evaluation and supervision on the public health care institution, and the business scope of the public health care institution mainly depends on the qualification authentication. The private for-profit/non-profit health care institution is under the government’s regulation, and the business scope of the private for-profit/non-profit health care institution mainly depends on the government’s judgment of the health care needs of the whole society.

6. Supervision/Monitoring: the setting of standards for both price and quality of health care and the work of addressing the potential market failures are mainly done by the government and partly done by the civic organization. The aim of the government’s supervision and monitoring is to achieve a guaranteed minimum outcome of health care provision, while the aim of the civic organization’s supervision and monitoring is to meet its social/organizational objectives.

7. Compensation choice: whether the government chooses to compensate the supply-side or the demand-side in the health care market depends on the type of health care, the government usually compensates the supply-side for basic health care, but the government usually compensates the demand-side for non-basic health care.

8. Health insurance system: a mandatory social health insurance system is currently implemented as the backbone of the medical security system, and it is responsible for the reimbursement of basic health care of the public. However, the non-mandatory multilevel public health insurance system that provides the customized insurance packages for the whole society will be implemented and will gradually replace the mandatory social health insurance system. Both the commercial health insurance system and the medical aid system that mainly consist of the private for-profit/non-profit institution are responsible for the reimbursement of non-basic health care of the insured person.

9. Financing: the financing of health care is composed of both public and private financing, here, public financing is mainly from both the central/local government and the state owned enterprise, and private financing is mainly from the private financial institution.

*Alternative approach 3*: *The market-oriented health care provider approach*

1. Basic health care: both the public health care institution and the private for-profit/non-profit health care institution can enter the basic health care market freely. The business scope of any type of health care institution mainly depends on the qualification authentication, and all types of health care institutions can compete in a fair environment under the government’s regulation.

2. Non-basic health care: all types of health care institutions can enter the non-basic health care market freely, and the business scope of any type of health care institution mainly depends on the competition result of a level playing field under the government’s regulation.

3. Health care and insurance system: the “internal market reform” is implemented and the publicly-funded health care system is replaced by the social health system. In fact, this process is similar to the internal market reform of the British National Health Service, the state designates a number of districts, and in each district a regulatory body is established to act as the buyer of health care services, and according to the population, age, disease type, morbidity, and other factors of the specific region, the national experts determine the allocation of health care expenses and appropriate funds to these regulatory bodies that are responsible for supplying both basic public health services and basic health care services to the public free of charge. A non-mandatory multilevel public health insurance system is implemented as the backbone of the medical security system, while both the medical aid system and the commercial health insurance system also play important roles in the health insurance system.

4. Medicine/medical device supply: the desired medicine/medical device is mainly supplied by the private for-profit institution and partly supplied by both the public interest institution and the private non-profit institution. The private for-profit institution’s incentives for medicine/medical device supply are making profit. Here, other major indicators such as medicine/medical device production facilities, R&D, production regulation, supply chain, and private medicine/medical device firms should also be assessed for this attribute.

5. Management system: the government exercises the functions of an independent regulator and treats both the public health care institution and the private for-profit/non-profit health care institution fairly. The business scope of any type of health care institution mainly depends on either the qualification authentication or the competition result of a level playing field.

6. Supervision/Monitoring: the setting of standards for both price and quality of health care and the work of addressing the potential market failures are mainly done by both the government and the civic organization. The aim of the government’s supervision and monitoring is to achieve a guaranteed minimum outcome of health care provision, while the aim of the civic organization’s supervision and monitoring is to meet its social/organizational objectives.

7. Compensation choice: the government mainly compensates the demand-side in both the basic health care market and the non-basic health care market. Specifically, the government not only provides health care subsidies for patients, but also provides health insurance subsidies for the poor.

8. Health insurance system: a non-mandatory public health insurance system is implemented as the backbone of the medical security system. In fact, this multilevel public health insurance system provides the customized insurance packages for the whole society, and it is responsible for the reimbursement of customized health care of the insured person. Both the commercial health insurance system and the medical aid system that mainly consist of the private for-profit/non-profit institution are responsible for the reimbursement of appropriate health care of the insured person.

9. Financing: the financing of health care mainly refers to private financing from the private financial institution.

#### Selection of criteria attributes

The overall goal for China’s institutional problem of health care provider selection can be separated into four sub-goals, and they are organized as criteria attributes, involving efficiency, economy, effectiveness, and equity, in the problem-solving process, each of them is treated as a separate hierarchy [36,37].

1. Efficiency: the efficiency attribute explores how medical resources are translated into health care. An efficient operation maximizes the health care provision for a given set of medical resources, or it minimizes the medical resources required to achieve a given goal of health care provision.

2. Economy: the economy attribute explores whether medical resources are acquired at the lowest cost and at the right time, and whether the method of producing the requisite health care is economical.

3. Effectiveness: the effectiveness attribute explores the extent to which the health care provision of the health care institution achieves the desired outcome.

4. Equity: the equity attribute explores whether health care is being provided impartially, fairly, and equitably, and reflects the extent to which the health care institution has achieved and has been able to maintain an equitable supply of comparable health care across demographic groups, regions, urban and rural areas, and so on.

#### Selection of strategic attributes

The following seven strategic attributes are the central attributes that link together activity attributes, regulatory attributes, and market attributes for the institutional problem of health care provider selection [37,38].

1. Market potential: the market potential attribute reflects the profitability of the health care market for the private health care institution. Both the regional natural monopoly of health care and the stable return on investment are the major incentives for the involvement of the private health care institution in health care provision.

2. Institutional guarantee: the institutional guarantee attribute reflects the extent to which the institutional and legal system, the government’s regulation, and the political will of the senior leadership inspire the confidence of the private health care institution and facilitate the efficient PPP operation. Unguaranteed institutional systems increase uncertainty and risk in both the private health care institution and the PPP operation, thus weakening the willingness of the private for-profit/non-profit health care institution to participate in health care provision.

3. Government credibility: the government credibility attribute mainly refers to how the government exercises its rights and supervision powers in alignment with laws and regulations. Government credibility is crucial for the private for-profit/non-profit investment in health care. The key contributors for guaranteeing government credibility include political checks and balances, an independent juridical system, and independent regulations. The absence of government credibility not only increases uncertainty and risk in both the private health care institution and the PPP operation, but also decreases the credibility of the public health care institution.

4. Financial accessibility: the financial accessibility attribute reflects the availability of both domestic and international capitals for both the private for-profit/non-profit health care institution and the public health care institution to finance a health care project. A mature financial market can help both the private for-profit/non-profit health care institution and the public health care institution raise funds at low cost and with low risk. A health care project with high financing costs inevitably leads to the expensive health care delivery and ultimately harms social welfare.

5. Government capacity: the government capacity attribute refers to the expertise, knowledge, and information that the government and the public health care institution have for negotiating, operating, and supervising all types of health care projects. Government capacity is a prerequisite for both the government and the public health care institution to initiate and manage a successful health care project, and this type of health care project in turn guarantees that the health care provision by the private for-profit/non-profit health care institution does not sacrifice either the public health care institution’s interests or social welfare.

6. Centralized management: the centralized management attribute reflects the centralization and unification of the government’s participation in health care provision, and it includes the negotiation, approval, enforcement, and supervision. A centralized regulatory framework is helpful for eliminating the conflicts among the public health care institutions and providing the private for-profit/non-profit health care institutions with a clear guideline for interacting with both the government and the public health care institutions.

7. Control of corruption: the control of corruption attribute indicates the ability of the government to prevent and control the corruption and lobbying. In our study, corruption is defined as the exercise of public power for private gain. Corruption and lobbies may speed up the approval and enforcement of the health care project, but they can destroy both the fair market competition and the private for-profit/non-profit investor’s confidence in the long run. In recent years this problem has become more and more serious in China and has greatly harmed social welfare, so it is worth the concern of the government and society as a whole.

### Data

In order to collect the data for determining the optimal solution to China’s institutional problem of health care provider selection from various experts, after both the general framework of China’s institutional problem of health care provider selection and the range of optional implementation methodologies were determined, this study collaborated with the National Bureau of Statistics of China to commission a large-scale 2009 to 2010 national expert survey through the organization of a pilot health care provider research system in 19 provinces, autonomous regions, and municipalities directly under the central government.

Under the support of the pilot health care provider research system, this survey adopted the two-stage probability proportional to size (PPS) systematic sampling technique to select a probability sample of 4,670 experts, and they were divided into seven categories: doctors; pharmacists; nurses; hospital administrators; health officials in health administration departments; officials in medical insurance agencies; and health care researchers in colleges, universities, and research departments. In this survey, the professional survey teams from the National Bureau of Statistics and local Bureaus of Statistics conducted the face-to-face interviews through the online information system of the pilot health care provider research system. The professional investigator usually first invited the selected expert to fill out the survey questionnaire in the online information system of the pilot health care provider research system, no replacement was made if the selected expert was away, refused to be interviewed, or failed to be interviewed after three attempts. If the selected expert agreed to fill out the survey questionnaire in the online information system of the pilot health care provider research system, but she/he was unavailable, or disabled in a way that impeded her/him from filling out the survey questionnaire, another expert that knew her/him best served as the respondent, and this expert was also asked to report her/his assessed values of the questions in the survey questionnaire through the online information system of the pilot health care provider research system in order to check bias. A total of 3,914 valid responses were generated in the 2009 to 2010 national expert survey; they included 490 doctors, 481 pharmacists, 479 nurses, 483 hospital administrators, 603 health officials in health administration departments, 598 officials in medical insurance agencies, and 780 health care researchers; the response rate was 83.81%.

The survey questionnaire consisted of three parts: the first inquired about the respondent’s assessed values of the composite weights of activity attributes, regulatory attributes, market attributes, and strategic attributes; the second inquired about the respondent’s assessed values of the relative importance of activity attributes, regulatory attributes, market attributes, and strategic attributes with respect to criteria attributes; the third inquired about the respondent’s assessed values of the relative preferences for criteria attributes of alternatives with respect to activity attributes, regulatory attributes, market attributes, and strategic attributes. The contents of the survey questionnaire were determined on the basis of both the general framework of China’s institutional problem of health care provider selection and the range of optional implementation methodologies.

The use of the dataset was approved by the National Bureau of Statistics of China.

### Selection of the implementation methodology

A large number of implementation methodologies such as the weighted point method [39], the matrix approach [40], the vendor performance matrix approach [41], and the vendor profile analysis [42] were adopted to evaluate the qualifications of health care providers. These implementation methodologies were easy to implement, but were overly simplistic, and the integration of both qualitative and quantitative factors remained a central problem in these implementation methodologies.

Many researchers usually adopted the more sophisticated multi-attribute decision methodologies in the complex provider selection problem, and these types of implementation methodologies involved the analytic hierarchy process (AHP) [43,44], the multi-objective programming [45], and the multi-attribute utility theory [46]. However, these earlier implementation methodologies did not explicitly address the interactions among decision factors. In the literature, up till now, such interactions could be explicitly addressed only using the analytic network process (ANP) [47-49].

The ANP, a general version of the AHP [50], could further explicitly address the interactions among attributes and sub-attributes in the complex provider selection problem [51-53]. The ANP evaluated the impact of the dependency among elements through the development of a “super matrix” that was likened to the Markov chain [52], and this characteristic made ANP the only implementation methodology that could address the inter-dependent nature of the decision attributes [54,55]. The strength of the ANP model was its ability to structure the attributes and the corresponding problems in a systematic manner that helped the decision maker discern the inter-related aspects of the system [56].

Since China’s institutional problem of health care provider selection required an implementation methodology that could handle possible interactions among decision factors/constraints in a multi-stage setting, so far in the literature only the ANP implementation methodology could best achieve this difficult goal. The ANP was then the optimal implementation methodology that could help the decision maker effectively promote various experts’ views into various optimal solutions to China’s institutional problem of health care provider selection.

### Detailed description of the ANP implementation methodology

After China’s institutional problem of health care provider selection was transformed to an ANP problem, efficiency, economy, effectiveness, and equity were treated as four separate hierarchies (also four sub-goals that constituted the overall goal) to determine the optimal health care provider approach in the alternative cluster. In order to solve the ANP formulation, the ANP problem for China’s institutional problem of health care provider selection was separated into two parts. In the first part the weights of activity attributes, regulatory attributes, market attributes, and strategic attributes were determined, and in the second part the weights of alternatives were determined. These two parts could be addressed in two phases, since the activity cluster, the regulatory cluster, the market cluster, and the strategic cluster did not depend on the alternative cluster.

Determining the weights of attributes:

A super matrix for each hierarchy was constructed as follows.

$$\begin{array}{l} \;\text{Goal}\;P_{h}\;C_{1}\;\cdots \;C_{i}\;\cdots \;C_{K}\\ W_{h}=\begin{array}{c} \text{Goal}\\ P_{h}\\ C_{1}\\ \vdots \\ C_{i}\\ \vdots \\ C_{K} \end{array}\left(\begin{array}{ccccccc} 0 & 0 & 0 & \cdots & 0 & \cdots & 0\\ 1 & 0 & 0 & \cdots & 0 & \cdots & 0\\ 0 & A_{\mathrm{PC}1} & A_{C11} & \cdots & A_{C1i} & \cdots & A_{C1K}\\ \vdots & \vdots & \vdots & & \vdots & & \vdots \\ 0 & A_{\text{PCi}} & A_{\mathrm{Ci}1} & \cdots & A_{\text{Cii}} & \cdots & A_{\text{CiK}}\\ \vdots & \vdots & \vdots & & \vdots & & \vdots \\ 0 & A_{\text{PCK}} & A_{\mathrm{CK}1} & \cdots & A_{\text{CKi}} & \cdots & A_{\text{CKK}} \end{array}\right) \end{array}$$

here *W*_*h*_ was the super matrix for hierarchy *h*, *h* = 1, 2, 3, and 4;

*P*_*h*_ was the criterion attribute, with *P*_*1*_ = (efficiency), *P*_*2*_ = (economy), *P*_*3*_ = (effectiveness), and *P*_*4*_ = (equity);

*C*_*i*_ represented the activity cluster, the regulatory cluster, the market cluster, and the strategic cluster, with *i* = 1, 2, 3, and 4, respectively;

*A*_*PCi*_ was the relative importance matrix of the cluster *C*_*i*_ with respect to the criterion attribute *P*_*h*_;

*A*_*Cij*_ was the relative importance matrix of the cluster *C*_*i*_ with respect to the cluster *C*_*j*_*.*

In order to derive the weights in the super matrix *W*_*h*_, pair wise comparisons between attributes were performed in response to the following question: which of the two attributes contributed more to the criterion attribute *P*_*h*_? Next, the clusters that were directly affected by other clusters were found, and the relative importance of these clusters to the leading cluster was determined. Within the super matrix framework, the relative importance of a column of clusters to the leading cluster in that column was determined. The weights of the relative importance of the clusters (in a column) were used to normalize the attributes of the respective clusters in that column. This maintained the unity property of the columns of the super matrix. The super matrix *W*_*h*_ was then raised to a large power. The relative weights of attributes with respect to the criterion attribute *P*_*h*_ were given in the second column of the converged matrix *W*_*h*_^∞^.

Determining the weights of alternatives:

The relative priorities of alternatives with respect to the criterion attribute *P*_*h*_ were obtained by:

$$Z_{h}=A_{h}{\;}_{*}\;M_{h}$$

here *A*_*h*_ was the matrix representing the relative priorities of alternatives with respect to their leading attributes for hierarchy *h*; and *M*_*h*_ was the vector representing the relative weights of attributes with respect to the criterion attribute *P*_*h*_, given in the second column of the converged matrix *W*_*h*_^∞^.

Next, the total outcome formula was used to combine the respective results in the separate hierarchies [56]. The top-ranked alternative for each hierarchy was rated and the four ratings were normalized. Let b, c, d, and e be the respective normalized ratings for efficiency, economy, effectiveness, and equity. In this study efficiency, economy, effectiveness, and equity were taken as equally important, then b, c, d, and e were set to the same value 0.25. The relative priorities of alternatives with respect to the overall goal were obtained by computing the value of b*Z*_*1*_ + c*Z*_*2*_ + d*Z*_*3*_ + e*Z*_*4*_. It should be noted that there were a variety of other approaches to aggregate the *Z*_*h*_’s for the hierarchies [56].

## Results

On the basis of the doctors’ average assessed values of the composite weights of activity attributes, regulatory attributes, market attributes, and strategic attributes in the national expert survey, the composite weights in the non-standardized super matrix of the ANP problem were calculated and prepared in the first table. The doctors’ average assessed values of the relative importance of activity attributes, regulatory attributes, market attributes, and strategic attributes with respect to criteria attributes in the national expert survey were prepared in the second table. Then the non-standardized super matrix in the first table was weighted for each hierarchy (refer to efficiency, economy, effectiveness, and equity) according to the weights in the second table, as the result, the limiting weighted super matrix for each hierarchy that incorporated both inner-dependency effects and inter-dependency effects was determined. The resulting weights of the attributes (given in the second column of the converged matrix *W*_*h*_^∞^) for each hierarchy were then found and prepared in the third table.

The doctors’ average assessed values of the relative preferences for criteria attributes of alternatives with respect to activity attributes, regulatory attributes, market attributes, and strategic attributes in the national expert survey were prepared in the fourth table. Through multiplying the third table and the fourth table, the weights of alternatives with respect to efficiency, economy, effectiveness, and equity were obtained and prepared in the fifth table. The top-ranked weights for efficiency, economy, effectiveness, and equity were then used to normalize the matrix in the fifth table. The resulting matrix for doctors is presented in Table 1.

**Table 1:** The resulting matrix for doctors

	**Efficiency**	**Economy**	**Effectiveness**	**Equity**	**Overall goal**
	***Z***_***1***_	***Z***_***2***_	***Z***_***3***_	***Z***_***4***_	**b*Z***_***1 ***_**+ c*Z***_***2 ***_**+ d*Z***_***3***_**+ e*Z***_***4***_
Alt 1	0.996	0.786	1.000	0.984	0.942
Alt 2	1.000	0.810	0.943	1.000	0.938
Alt 3	0.959	1.000	0.913	0.936	0.952

Here b = c = d = e = 0.25.

On the basis of Table 1, from the doctors’ point of view, the PPP approach (Alt 2) was the optimal approach in terms of efficiency and equity; the market-oriented health care provider approach (Alt 3) was the optimal approach in terms of economy; the traditional government’s regulation-oriented health care provider approach (Alt 1) was the optimal approach in terms of effectiveness; the market-oriented health care provider approach (Alt 3) was the optimal approach for the overall goal, the traditional government’s regulation-oriented health care provider approach (Alt 1) was the second-best approach for the overall goal, and the PPP approach (Alt 2) was the third-best approach for the overall goal.

Since the problem-solving processes for pharmacists, nurses, hospital administrators, health officials in health administration departments, officials in medical insurance agencies, and health care researchers were similar to the above problem-solving process for doctors, only the resulting matrix for each category of experts was simply presented as follows.

The resulting matrix for pharmacists is presented in Table 2. On the basis of Table 2, from the pharmacists’ point of view, the traditional government’s regulation-oriented health care provider approach (Alt 1) was the optimal approach in terms of efficiency, effectiveness, and equity; the market-oriented health care provider approach (Alt 3) was the optimal approach in terms of economy; the traditional government’s regulation-oriented health care provider approach (Alt 1) was the optimal approach for the overall goal, the market-oriented health care provider approach (Alt 3) was the second-best approach for the overall goal, and the PPP approach (Alt 2) was the third-best approach for the overall goal.

**Table 2:** The resulting matrix for pharmacists

	**Efficiency**	**Economy**	**Effectiveness**	**Equity**	**Overall goal**
	***Z***_***1***_	***Z***_***2***_	***Z***_***3***_	***Z***_***4***_	**b*Z***_***1 ***_**+ c*Z***_***2 ***_**+ d*Z***_***3 ***_**+ e*Z***_***4***_
Alt 1	1.000	0.823	1.000	1.000	0.956
Alt 2	0.994	0.829	0.900	0.973	0.924
Alt 3	0.943	1.000	0.926	0.910	0.945

Here b = c = d = e = 0.25.

The resulting matrix for nurses is presented in Table 3. On the basis of Table 3, from the nurses’ point of view, the PPP approach (Alt 2) was the optimal approach in terms of efficiency; the market-oriented health care provider approach (Alt 3) was the optimal approach in terms of economy; the traditional government’s regulation-oriented health care provider approach (Alt 1) was the optimal approach in terms of effectiveness and equity; the PPP approach (Alt 2) was the optimal approach for the overall goal, the market-oriented health care provider approach (Alt 3) was the second-best approach for the overall goal, and the traditional government’s regulation-oriented health care provider approach (Alt 1) was the third-best approach for the overall goal.

**Table 3:** The resulting matrix for nurses

	**Efficiency**	**Economy**	**Effectiveness**	**Equity**	**Overall goal**
	***Z***_***1***_	***Z***_***2***_	***Z***_***3***_	***Z***_***4***_	**b*Z***_***1 ***_+ **c*Z***_***2 ***_+ **d*Z***_***3 ***_+ **e*Z***_***4***_
Alt 1	0.904	0.734	1.000	1.000	0.909
Alt 2	1.000	0.785	0.905	0.991	0.920
Alt 3	0.977	1.000	0.911	0.787	0.919

Here b = c = d = e = 0.25.

The resulting matrix for hospital administrators is presented in Table 4. On the basis of Table 4, from the hospital administrators’ point of view, the PPP approach (Alt 2) was the optimal approach in terms of efficiency; the market-oriented health care provider approach (Alt 3) was the optimal approach in terms of economy; the traditional government’s regulation-oriented health care provider approach (Alt 1) was the optimal approach in terms of effectiveness and equity; the traditional government’s regulation-oriented health care provider approach (Alt 1) was the optimal approach for the overall goal, the market-oriented health care provider approach (Alt 3) was the second-best approach for the overall goal, and the PPP approach (Alt 2) was the third-best approach for the overall goal.

**Table 4:** The resulting matrix for hospital administrators

	**Efficiency**	**Economy**	**Effectiveness**	**Equity**	**Overall goal**
	***Z***_***1***_	***Z***_***2***_	***Z***_***3***_	***Z***_***4***_	**b*Z***_***1 ***_**+ c*Z***_***2 ***_**+ d*Z***_***3 ***_**+ e*Z***_***4***_
Alt 1	0.962	0.766	1.000	1.000	0.932
Alt 2	1.000	0.798	0.876	0.960	0.908
Alt 3	0.909	1.000	0.876	0.920	0.926

Here b = c = d = e = 0.25.

The resulting matrix for health officials in health administration departments is presented in Table 5. On the basis of Table 5, from the point of view of health officials in health administration departments, the traditional government’s regulation-oriented health care provider approach (Alt 1) was the optimal approach in terms of efficiency and equity; the market-oriented health care provider approach (Alt 3) was the optimal approach in terms of economy and effectiveness; the traditional government’s regulation-oriented health care provider approach (Alt 1) was the optimal approach for the overall goal, the market-oriented health care provider approach (Alt 3) was the second-best approach for the overall goal, and the PPP approach (Alt 2) was the third-best approach for the overall goal.

**Table 5:** The resulting matrix for health officials in health administration departments

	**Efficiency**	**Economy**	**Effectiveness**	**Equity**	**Overall goal**
	***Z***_***1***_	***Z***_***2***_	***Z***_***3***_	***Z***_***4***_	**b*Z***_***1 ***_**+ c*Z***_***2 ***_**+ d*Z***_***3 ***_**+ e*Z***_***4***_
Alt 1	1.000	0.876	0.997	1.000	0.968
Alt 2	0.993	0.904	0.925	0.878	0.925
Alt 3	0.918	1.000	1.000	0.903	0.955

Here b = c = d = e = 0.25.

The resulting matrix for officials in medical insurance agencies is presented in Table 6. On the basis of Table 6, from the point of view of officials in medical insurance agencies, the PPP approach (Alt 2) was the optimal approach in terms of efficiency; the market-oriented health care provider approach (Alt 3) was the optimal approach in terms of economy; the traditional government’s regulation-oriented health care provider approach (Alt 1) was the optimal approach in terms of effectiveness and equity; the PPP approach (Alt 2) was the optimal approach for the overall goal, the market-oriented health care provider approach (Alt 3) was the second-best approach for the overall goal, and the traditional government’s regulation-oriented health care provider approach (Alt 1) was the third-best approach for the overall goal.

**Table 6:** The resulting matrix for officials in medical insurance agencies

	**Efficiency**	**Economy**	**Effectiveness**	**Equity**	**Overall goal**
	***Z***_***1***_	***Z***_***2***_	***Z***_***3***_	***Z***_***4***_	**b*Z***_***1 ***_**+ c*Z***_***2 ***_**+ d*Z***_***3 ***_**+ e*Z***_***4***_
Alt 1	0.803	0.667	1.000	1.000	0.868
Alt 2	1.000	0.859	0.871	0.996	0.931
Alt 3	0.815	1.000	0.753	0.976	0.886

Here b = c = d = e = 0.25.

The resulting matrix for health care researchers is presented in Table 7. On the basis of Table 7, from the health care researchers’ point of view, the market-oriented health care provider approach (Alt 3) was the optimal approach in terms of efficiency and economy; the traditional government’s regulation-oriented health care provider approach (Alt 1) was the optimal approach in terms of effectiveness; the PPP approach (Alt 2) was the optimal approach in terms of equity; the PPP approach (Alt 2) was the optimal approach for the overall goal, the market-oriented health care provider approach (Alt 3) was the second-best approach for the overall goal, and the traditional government’s regulation-oriented health care provider approach (Alt 1) was the third-best approach for the overall goal.

**Table 7:** The resulting matrix for health care researchers

	**Efficiency**	**Economy**	**Effectiveness**	**Equity**	**Overall goal**
	***Z***_***1***_	***Z***_***2***_	***Z***_***3***_	***Z***_***4***_	**b*Z***_***1 ***_**+ c*Z***_***2 ***_**+ d*Z***_***3 ***_**+ e*Z***_***4***_
Alt 1	0.971	0.832	1.000	0.979	0.945
Alt 2	0.999	0.907	0.941	1.000	0.962
Alt 3	1.000	1.000	0.880	0.921	0.950

Here b = c = d = e = 0.25.

## Discussion

### Sensitivity tests of criteria attributes

In this study, four sub-goals of China’s institutional problem of health care provider selection involving efficiency, economy, effectiveness, and equity were taken as equally important. But in the reality, the decision maker usually needed to focus on the most important current sub-goals, then the sub-goals of efficiency, economy, effectiveness, and equity were usually taken as unequally important, and the respective normalized ratings for efficiency, economy, effectiveness, and equity were usually different. So this study further underwent sensitivity tests of criteria attributes in order to explore to what extent the weights of criteria attributes influenced the optimal solution to China’s institutional problem of health care provider selection.

In the sensitivity tests of criteria attributes, the test interval for the weight of each criterion attribute was chosen to be from 0.00 to 1.00, and it was stipulated that if the weight of the target criterion attribute was x, then the weights of the other three criteria attributes were set to (1-x)/3.

**Figure 2:**
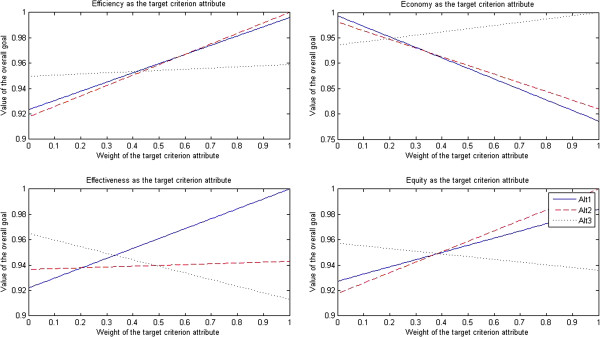
Sensitivity tests for doctors.

The results of sensitivity tests for doctors are presented in Figure 2.

After efficiency was selected as the target criterion attribute, when the weight of efficiency attribute dropped to less than 0.41, the market-oriented health care provider approach (Alt 3) turned into the optimal approach; when the weight of efficiency attribute was in the interval [0.42, 0.58], the traditional government’s regulation-oriented health care provider approach (Alt 1) turned into the optimal approach; when the weight of efficiency attribute rose to more than 0.59, the PPP approach (Alt 2) turned into the optimal approach.

After economy was selected as the target criterion attribute, when the weight of economy attribute dropped to less than 0.21, the traditional government’s regulation-oriented health care provider approach (Alt 1) turned into the optimal approach; when the weight of economy attribute rose to more than 0.22, the market-oriented health care provider approach (Alt 3) turned into the optimal approach; however, the PPP approach (Alt 2) was always the non-optimal approach.

After effectiveness was selected as the target criterion attribute, when the weight of effectiveness attribute dropped to less than 0.33, the market-oriented health care provider approach (Alt 3) turned into the optimal approach; when the weight of effectiveness attribute rose to more than 0.34, the traditional government’s regulation-oriented health care provider approach (Alt 1) turned into the optimal approach; however, the PPP approach (Alt 2) was always the non-optimal approach.

After equity was selected as the target criterion attribute, when the weight of equity attribute dropped to less than 0.38, the market-oriented health care provider approach (Alt 3) turned into the optimal approach; when the weight of equity attribute rose to more than 0.39, the PPP approach (Alt 2) turned into the optimal approach; however, the traditional government’s regulation-oriented health care provider approach (Alt 1) was always the non-optimal approach.

**Figure 3:**
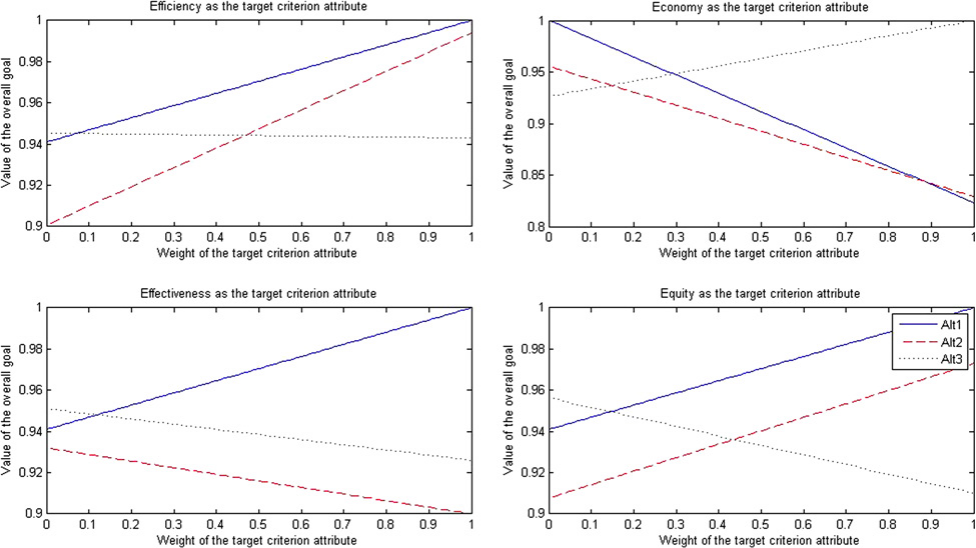
Sensitivity tests for pharmacists.

The results of sensitivity tests for pharmacists are presented in Figure 3.

After efficiency was selected as the target criterion attribute, when the weight of efficiency attribute dropped to less than 0.07, the market-oriented health care provider approach (Alt 3) turned into the optimal approach; when the weight of efficiency attribute rose to more than 0.08, the traditional government’s regulation-oriented health care provider approach (Alt 1) turned into the optimal approach; however, the PPP approach (Alt 2) was always the non-optimal approach.

After economy was selected as the target criterion attribute, when the weight of economy attribute dropped to less than 0.29, the traditional government’s regulation-oriented health care provider approach (Alt 1) turned into the optimal approach; when the weight of economy attribute rose to more than 0.30, the market-oriented health care provider approach (Alt 3) turned into the optimal approach; however, the PPP approach (Alt 2) was always the non-optimal approach.

After effectiveness was selected as the target criterion attribute, when the weight of effectiveness attribute dropped to less than 0.11, the market-oriented health care provider approach (Alt 3) turned into the optimal approach; when the weight of effectiveness attribute rose to more than 0.12, the traditional government’s regulation-oriented health care provider approach (Alt 1) turned into the optimal approach; however, the PPP approach (Alt 2) was always the non-optimal approach.

After equity was selected as the target criterion attribute, when the weight of equity attribute dropped to less than 0.14, the market-oriented health care provider approach (Alt 3) turned into the optimal approach; when the weight of equity attribute rose to more than 0.15, the traditional government’s regulation-oriented health care provider approach (Alt 1) turned into the optimal approach; however, the PPP approach (Alt 2) was always the non-optimal approach.

**Figure 4:**
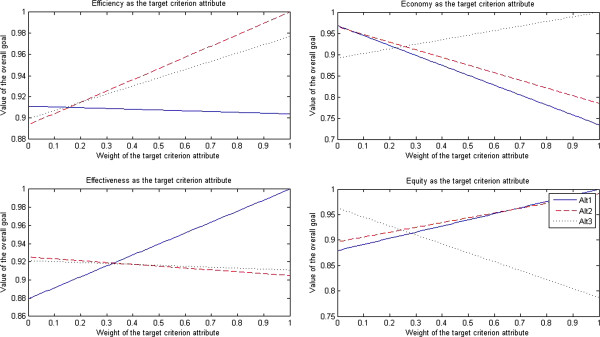
Sensitivity tests for nurses.

The results of sensitivity tests for nurses are presented in Figure 4.

After efficiency was selected as the target criterion attribute, when the weight of efficiency attribute dropped to less than 0.14, the traditional government’s regulation-oriented health care provider approach (Alt 1) turned into the optimal approach; when the weight of efficiency attribute was in the interval [0.15, 0.19], the market-oriented health care provider approach (Alt 3) turned into the optimal approach; when the weight of efficiency attribute rose to more than 0.20, the PPP approach (Alt 2) turned into the optimal approach.

After economy was selected as the target criterion attribute, when the weight of economy attribute dropped to less than 0.04, the traditional government’s regulation-oriented health care provider approach (Alt 1) turned into the optimal approach; when the weight of economy attribute was in the interval [0.05, 0.25], the PPP approach (Alt 2) turned into the optimal approach; when the weight of economy attribute rose to more than 0.26, the market-oriented health care provider approach (Alt 3) turned into the optimal approach.

After effectiveness was selected as the target criterion attribute, when the weight of effectiveness attribute dropped to less than 0.32, the PPP approach (Alt 2) turned into the optimal approach; when the weight of effectiveness attribute rose to more than 0.33, the traditional government’s regulation-oriented health care provider approach (Alt 1) turned into the optimal approach; however, the market-oriented health care provider approach (Alt 3) was always the non-optimal approach.

After equity was selected as the target criterion attribute, when the weight of equity attribute dropped to less than 0.24, the market-oriented health care provider approach (Alt 3) turned into the optimal approach; when the weight of equity attribute was in the interval [0.25, 0.65], the PPP approach (Alt 2) turned into the optimal approach; when the weight of equity attribute rose to more than 0.66, the traditional government’s regulation-oriented health care provider approach (Alt 1) turned into the optimal approach.

**Figure 5:**
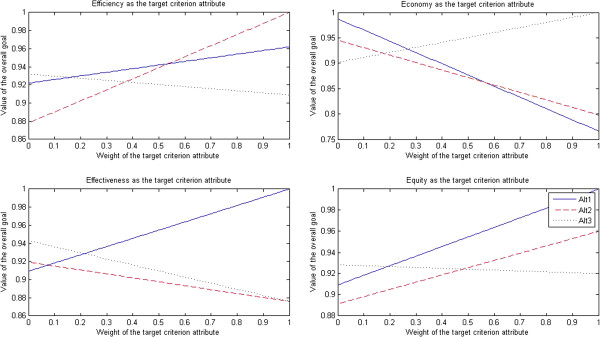
Sensitivity tests for hospital administrators.

The results of sensitivity tests for hospital administrators are presented in Figure 5.

After efficiency was selected as the target criterion attribute, when the weight of efficiency attribute dropped to less than 0.15, the market-oriented health care provider approach (Alt 3) turned into the optimal approach; when the weight of efficiency attribute was in the interval [0.16, 0.53], the traditional government’s regulation-oriented health care provider approach (Alt 1) turned into the optimal approach; when the weight of efficiency attribute rose to more than 0.54, the PPP approach (Alt 2) turned into the optimal approach.

After economy was selected as the target criterion attribute, when the weight of economy attribute dropped to less than 0.26, the traditional government’s regulation-oriented health care provider approach (Alt 1) turned into the optimal approach; when the weight of economy attribute rose to more than 0.27, the market-oriented health care provider approach (Alt 3) turned into the optimal approach; however, the PPP approach (Alt 2) was always the non-optimal approach.

After effectiveness was selected as the target criterion attribute, when the weight of effectiveness attribute dropped to less than 0.21, the market-oriented health care provider approach (Alt 3) turned into the optimal approach; when the weight of effectiveness attribute rose to more than 0.22, the traditional government’s regulation-oriented health care provider approach (Alt 1) turned into the optimal approach; however, the PPP approach (Alt 2) was always the non-optimal approach.

After equity was selected as the target criterion attribute, when the weight of equity attribute dropped to less than 0.19, the market-oriented health care provider approach (Alt 3) turned into the optimal approach; when the weight of equity attribute rose to more than 0.20, the traditional government’s regulation-oriented health care provider approach (Alt 1) turned into the optimal approach; however, the PPP approach (Alt 2) was always the non-optimal approach.

**Figure 6:**
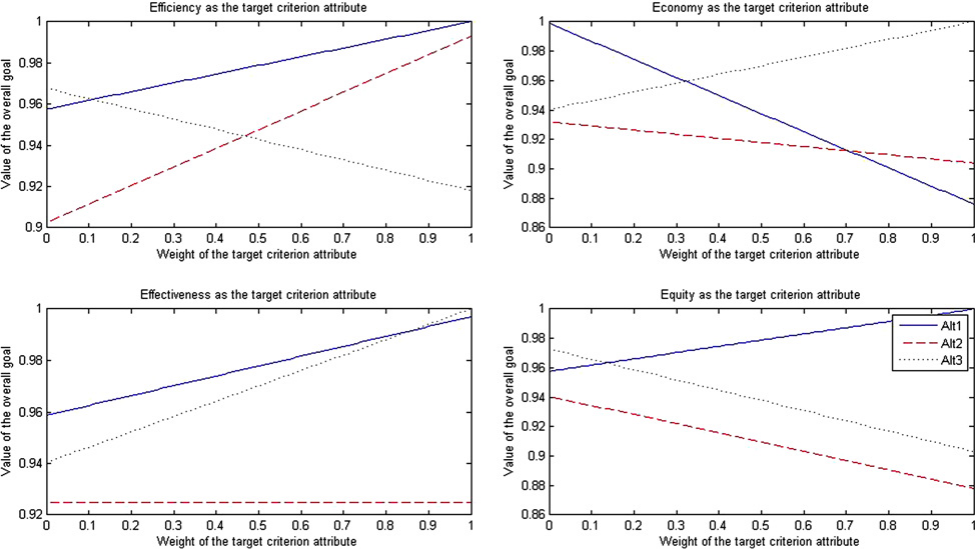
Sensitivity tests for health officials in health administration departments.

The results of sensitivity tests for health officials in health administration departments are presented in Figure 6.

After efficiency was selected as the target criterion attribute, when the weight of efficiency attribute dropped to less than 0.10, the market-oriented health care provider approach (Alt 3) turned into the optimal approach; when the weight of efficiency attribute rose to more than 0.11, the traditional government’s regulation-oriented health care provider approach (Alt 1) turned into the optimal approach; however, the PPP approach (Alt 2) was always the non-optimal approach.

After economy was selected as the target criterion attribute, when the weight of economy attribute dropped to less than 0.32, the traditional government’s regulation-oriented health care provider approach (Alt 1) turned into the optimal approach; when the weight of economy attribute rose to more than 0.33, the market-oriented health care provider approach (Alt 3) turned into the optimal approach; however, the PPP approach (Alt 2) was always the non-optimal approach.

After effectiveness was selected as the target criterion attribute, when the weight of effectiveness attribute dropped to less than 0.85, the traditional government’s regulation-oriented health care provider approach (Alt 1) turned into the optimal approach; when the weight of effectiveness attribute rose to more than 0.86, the market-oriented health care provider approach (Alt 3) turned into the optimal approach; however, the PPP approach (Alt 2) was always the non-optimal approach.

After equity was selected as the target criterion attribute, when the weight of equity attribute dropped to less than 0.13, the market-oriented health care provider approach (Alt 3) turned into the optimal approach; when the weight of equity attribute rose to more than 0.14, the traditional government’s regulation-oriented health care provider approach (Alt 1) turned into the optimal approach; however, the PPP approach (Alt 2) was always the non-optimal approach.

**Figure 7:**
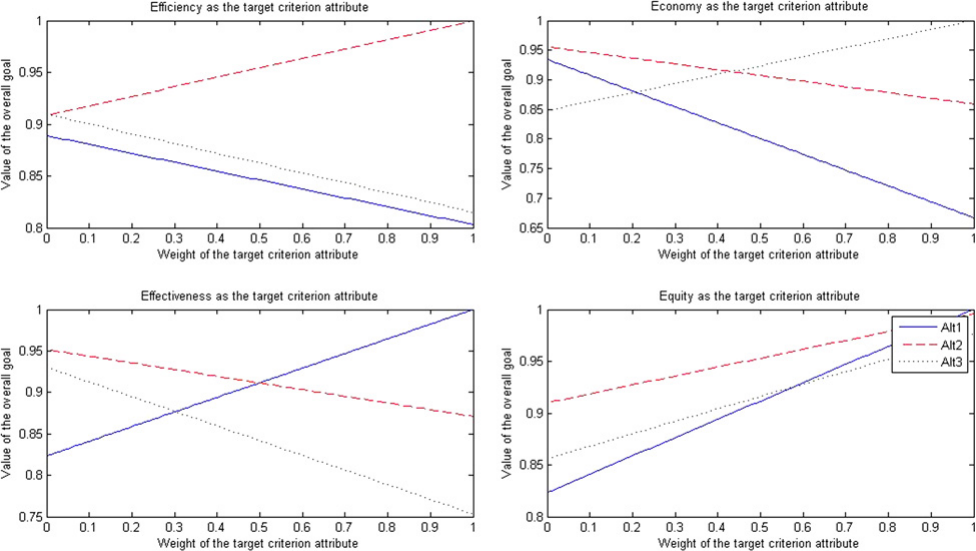
Sensitivity tests for officials in medical insurance agencies.

The results of sensitivity tests for officials in medical insurance agencies are presented in Figure 7.

After efficiency was selected as the target criterion attribute, when the weight of efficiency attribute dropped to less than 0.005, the market-oriented health care provider approach (Alt 3) turned into the optimal approach; when the weight of efficiency attribute rose to more than 0.01, the PPP approach (Alt 2) turned into the optimal approach; however, the traditional government’s regulation-oriented health care provider approach (Alt 1) was always the non-optimal approach.

After economy was selected as the target criterion attribute, when the weight of economy attribute dropped to less than 0.43, the PPP approach (Alt 2) turned into the optimal approach; when the weight of economy attribute rose to more than 0.44, the market-oriented health care provider approach (Alt 3) turned into the optimal approach; however, the traditional government’s regulation-oriented health care provider approach (Alt 1) was always the non-optimal approach.

After effectiveness was selected as the target criterion attribute, when the weight of effectiveness attribute dropped to less than 0.49, the PPP approach (Alt 2) turned into the optimal approach; when the weight of effectiveness attribute rose to more than 0.50, the traditional government’s regulation-oriented health care provider approach (Alt 1) turned into the optimal approach; however, the market-oriented health care provider approach (Alt 3) was always the non-optimal approach.

After equity was selected as the target criterion attribute, when the weight of equity attribute dropped to less than 0.95, the PPP approach (Alt 2) turned into the optimal approach; when the weight of equity attribute rose to more than 0.96, the traditional government’s regulation-oriented health care provider approach (Alt 1) turned into the optimal approach; however, the market-oriented health care provider approach (Alt 3) was always the non-optimal approach.

**Figure 8:**
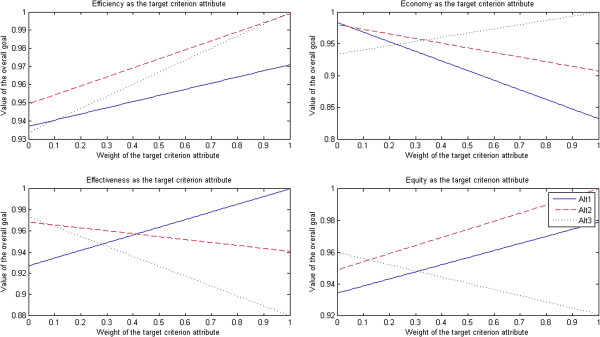
Sensitivity tests for health care researchers.

The results of sensitivity tests for health care researchers are presented in Figure 8.

After efficiency was selected as the target criterion attribute, when the weight of efficiency attribute dropped to less than 0.94, the PPP approach (Alt 2) turned into the optimal approach; when the weight of efficiency attribute rose to more than 0.94, the market-oriented health care provider approach (Alt 3) turned into the optimal approach; however, the traditional government’s regulation-oriented health care provider approach (Alt 1) was always the non-optimal approach.

After economy was selected as the target criterion attribute, when the weight of economy attribute dropped to less than 0.04, the traditional government’s regulation-oriented health care provider approach (Alt 1) turned into the optimal approach; when the weight of economy attribute was in the interval [0.05, 0.33], the PPP approach (Alt 2) turned into the optimal approach; when the weight of economy attribute rose to more than 0.34, the market-oriented health care provider approach (Alt 3) turned into the optimal approach.

After effectiveness was selected as the target criterion attribute, when the weight of effectiveness attribute dropped to less than 0.07, the market-oriented health care provider approach (Alt 3) turned into the optimal approach; when the weight of effectiveness attribute was in the interval [0.08, 0.41], the PPP approach (Alt 2) turned into the optimal approach; when the weight of effectiveness attribute rose to more than 0.42, the traditional government’s regulation-oriented health care provider approach (Alt 1) turned into the optimal approach.

After equity was selected as the target criterion attribute, when the weight of equity attribute dropped to less than 0.12, the market-oriented health care provider approach (Alt 3) turned into the optimal approach; when the weight of equity attribute rose to more than 0.13, the PPP approach (Alt 2) turned into the optimal approach; however, the traditional government’s regulation-oriented health care provider approach (Alt 1) was always the non-optimal approach.

In summary, the results of sensitivity tests for any category of experts revealed that the optimal solution to China’s institutional problem of health care provider selection was sensitive to the respective normalized ratings for the sub-goals of efficiency, economy, effectiveness, and equity.

### Limitations of this study

Although this study not only organized a pilot health care provider research system to collect the large-scale data for determining the optimal solution to China’s institutional problem of health care provider selection from various experts for the first time in China, but also applied the ANP implementation methodology to help the decision maker effectively promote various experts’ views into various optimal solutions to China’s institutional problem of health care provider selection on the basis of this large-scale data, it would still be worthwhile to apply the ANP implementation methodology to help the decision maker solve China’s institutional problem of health care provider selection through the organization of a formal health care provider research system in further research. The further research should also use a comprehensive sensitivity analysis to examine the significance of the weights of criteria attributes with respect to the decision for the selection of health care providers, on the basis of such a sensitivity analysis the decision maker could better understand to what extent the respective normalized ratings for the sub-goals of efficiency, economy, effectiveness, and equity influenced the optimal solution to China’s institutional problem of health care provider selection. If a new implementation methodology that could better handle possible interactions among decision factors/constraints in a multi-stage setting would be developed in future, it would also be worthwhile to apply this implementation methodology to help the decision maker effectively promote various experts’ views into various optimal solutions to China’s institutional problem of health care provider selection through the organization of the health care provider research system.

## Conclusions

Under the support of the ANP implementation methodology that could best handle the possible interactions among decision factors/constraints in the multi-stage setting in China’s institutional problem of health care provider selection, the data collected through a pilot health care provider research system in the 2009 to 2010 national expert survey could help the decision maker effectively promote various experts’ views into various optimal solutions to China’s institutional problem of health care provider selection.

After China’s institutional problem of health care provider selection was transformed to an ANP problem, through analyzing the dataset from the 2009 to 2010 national expert survey, this study obtained the following main results: the market-oriented health care provider approach was the optimal solution to China’s institutional problem of health care provider selection from the doctors’ point of view; the traditional government’s regulation-oriented health care provider approach was the optimal solution to China’s institutional problem of health care provider selection from the pharmacists’ point of view, the hospital administrators’ point of view, and the point of view of health officials in health administration departments; the PPP approach was the optimal solution to China’s institutional problem of health care provider selection from the nurses’ point of view, the point of view of officials in medical insurance agencies, and the health care researchers’ point of view.

The sensitivity tests revealed that the optimal solution to China’s institutional problem of health care provider selection was sensitive to the respective normalized ratings for the sub-goals of efficiency, economy, effectiveness, and equity.

## Abbreviations

AHP: Analytic hierarchy process; Alt 1: Traditional government’s regulation-oriented health care provider approach; Alt 2: PPP approach; Alt 3: Market-oriented health care provider approach; ANP: Analytic network process; PPP: Public private partnership; PPS: Probability proportional to size.

## Competing interests

The author declares that he has no competing interests.

## References

[B1] China National Health Economics Institute China National Health Account Report 2007 Beijing: Chinese Ministry of Health

[B2] Ministry of Health China Health Statistical Yearbook 2008 2008 Beijing: Peking Union Medical College Press

[B3] Tang L The influences of patient’s trust in medical service and attitude towards health policy on patient’s overall satisfaction with medical service and sub satisfaction in China BMC Public Health 2011 11 472 10.1186/1471-2458-11-472 21676228 PMC3129314

[B4] Tang L The influences of patient’s satisfaction with medical service delivery, assessment of medical service, and trust in health delivery system on patient’s life satisfaction in China Health Qual Life Outcomes 2012 10 111 10.1186/1477-7525-10-111 22978432 PMC3487997

[B5] Tang L The patient’s anxiety before seeing a doctor and her/his hospital choice behavior in China BMC Public Health 2012 12 1121 10.1186/1471-2458-12-1121 23270526 PMC3536590

[B6] Tang L China’s regional inequity in pharmacist’s drug safety practice Int J Equity Health 2012 11 38 10.1186/1475-9276-11-38 22867000 PMC3485099

[B7] Command-and-control versus regulated marketization: a review of eight proposals for China’s new health care reforms [http://www.chinahealthreform.org], [https://groups.google.com/forum/?fromgroups#!forum/crcpp], [http://www.cser.org.cn]

[B8] Hu S Models of the cooperative medical scheme Chin Prim Health 2003 17 1 5

[B9] Fang Y Guo A Li J Wang Y Hu S Zhou X Liang W National survey of community health services Chin Gen Pract 2005 17 1389 1395

[B10] Gao Q Ministry of Health’s Report on China's Healthcare System and Reform 2005 Beijing: Chinese Ministry of Health

[B11] Wang S Qin Q Tang Z Accumulating deposit: a way of individual premium collection in the new rural cooperative medical scheme Chin Health Econ 2007 26 38 40

[B12] Ahlfors UG Lewander T Lindström E Malt UF Lublin H Malm U Assessment of patient satisfaction with psychiatric care development and clinical evaluation of a brief consumer satisfaction rating scale (UKU-ConSat) Nord J Psychiat 2001 55 Suppl 44 71 90 10.1080/080394801317084437 11860667

[B13] Cai R The Reform of Health Security System in China 1998 Beijing: Ren Shi Publishers

[B14] Yip WC Wang H Liu Y Determinants of patient choice of medical provider: a case study in rural China Health Policy Plann 1998 13 3 311 322 10.1093/heapol/13.3.311 10187600

[B15] Ren R Zhang L Analysis of cooperative medical system in China: causes of success and failure and effect factors Chin Health Econ 2001 19 21 26

[B16] Lim MK Yang H Zhang T Feng W Zhou Z Public perceptions of private health care in socialist China Health Affair 2004 23 6 222 234 10.1377/hlthaff.23.6.222 15537602

[B17] Blumenthal D Hsiao W Privatization and its discontents: the evolving Chinese health care system New Engl J Med 2005 353 11 1165 1170 10.1056/NEJMhpr051133 16162889

[B18] Liu Y Bermana P Yip W Liang H Meng Q Qu J Li Z Health care in China: the role of non-government providers Health Policy 2006 77 2 212 220 10.1016/j.healthpol.2005.07.002 16112771

[B19] Hall JA Dornan MC Patient sociodemographic characteristics as predictors of satisfaction with medical care: a meta-analysis Soc Sci Med 1990 30 7 811 818 10.1016/0277-9536(90)90205-7 2138357

[B20] Hackman A Brown C Yang Y Goldberg R Kreyenbuhl J Lucksted A Wohlheiter K Dixon L Consumer satisfaction with inpatient psychiatric treatment among persons with severe mental illness Community Ment Health J 2007 43 6 551 564 10.1007/s10597-007-9098-3 17641972

[B21] Jiang L Gan C Kao B Zhang Y Zhang H Cai L Consumer satisfaction with public health care in China J Soc Sci 2009 5 3 223 235

[B22] Liu Y Rao K Wu J Gakidou E China's health system performance Lancet 2008 372 9653 1914 1923 10.1016/S0140-6736(08)61362-8 18930536

[B23] Sharan P Gallo C Gureje O Lamberte E Mari JJ Mazzotti G Patel V Swartz L Olifson S Levav I de Francisco A Saxena S Mental health research priorities in low- and middle-income countries of Africa, Asia, Latin America and the Caribbean Br J Psychiatry 2009 195 4 354 363 10.1192/bjp.bp.108.050187 19794206 PMC3432479

[B24] Lavis JN Guindon GE Cameron D Boupha B Dejman M Osei EJ Sadana R Bridging the gaps between research, policy and practice in low- and middle-income countries: a survey of researchers CMAJ 2010 182 9 E350 E361 20439449 10.1503/cmaj.081164 PMC2882466

[B25] Liu D Wang X Pan F Yang P Xu Y Tang X Hu J Rao K Harmonization of health data at national level: a pilot study in China Int J Med Inform 2010 79 6 450 458 10.1016/j.ijmedinf.2010.03.002 20399139

[B26] Zhao J Zhang Z Guo H Li Y Xue W Ren L Chen Y Chen S Zhang X E-health in China: challenges, initial directions, and experience Telemed E-Health 2010 16 3 344 349 10.1089/tmj.2009.0076 20406121

[B27] Liu S Zhou B Xie G Mei J Liu H Liu C Qi L Beyond regional health information exchange in China: a practical and industrial-strength approach AMIA Annu Symp Proc 2011 2011 824 833 22195140 PMC3243277

[B28] Gong P Liang S Carlton EJ Jiang Q Wu J Wang L Remais JV Urbanisation and health in China Lancet 2012 379 9818 843 852 10.1016/S0140-6736(11)61878-3 22386037 PMC3733467

[B29] Chen Y Lee J Analysis and Evaluation about the Barriers of the Adoption and Implementation of Electronic Health Record System: A Comparison Study between China and Korea 2012 Hokodate, Hokkaido: International Symposium on Information Technology in Medicine and Education (ITME)

[B30] Barney J Firm resources and sustained competitive advantage J Manage 1991 17 1 99 120

[B31] Gulati R Nohria N Zaheer A Strategic networks Strategic Manage J 2000 21 Special Issue 203 215

[B32] Daugherty PJ Stank TP Rogers DS Third-party logistics service providers: purchasers’ perceptions J Supply Chain Manage 1996 32 2 23 29 10.1111/j.1745-493X.1996.tb00222.x

[B33] Murphy PR Poist RF Third-party logistics: some user versus provider perspectives J Bus Logist 2000 21 1 121 133

[B34] Lai K Service capability and performance of logistics service providers Transp Res Part E: Logist Trans Rev 2004 40 5 385 399 10.1016/j.tre.2004.01.002

[B35] Jütting J PPP and Social Protection in Developing Countries: The Case of the Health Sector 1999 Geneva: The ILO workshop on “The extension of social protection”

[B36] Reider R Improving the Economy, Efficiency, and Effectiveness of Not-for-Profits: Conducting Operational Reviews 2001 USA: Wiley, John & Sons, Incorporated

[B37] Urio P Reconciling State, Market and Civil Society in China: The Long March Toward Prosperity 2008 London: Routledge Contemporary China Series

[B38] Yang Y Hou Y Wang Y Zhang W An Empirical Investigation of Critical PPP Factors in Transition Countries using Exploratory Factor Analysis 2010 Montreal: The 2010 Academy of Management Annual Meeting

[B39] Timmerman E An approach to vendor performance evaluation J Purchasing Mater Manage 1986 22 4 2 8

[B40] Gregory RE Source selection: a matrix approach J Purchasing Mater Manage 1986 22 2 24 29

[B41] Soukup WR Supplier selection strategies J Purchasing Mater Manage 1987 23 2 7 12

[B42] Thompson KN Supplier profile analysis J Purchasing Mater Manage 1990 26 1 11 18

[B43] Narasimhan R An analytical approach to supplier selection J Purchasing Mater Manage 1983 19 4 27 32

[B44] Nydick RL Hill RP Using the analytic hierarchy process to structure the supplier selection procedure J Purchasing Mater Manage 1992 25 2 31 36

[B45] Weber CA Ellram LM Supplier selection using multi-objective programming: a decision support system approach Int J Phys Distrib Logist Manage 1993 23 2 3 15 10.1108/09600039310038161

[B46] Min H International supplier selection: a multi-attribute utility approach Int J Phys Distrib Logist Manage 1994 24 5 24 33 10.1108/09600039410064008

[B47] Sarkis J Talluri S A model for strategic supplier selection J Supply Chain Manage 2002 38 1 18 28 10.1111/j.1745-493X.2002.tb00117.x

[B48] Bayazit O Use of analytic network process in vendor selection decisions Benchmark Int J 2006 13 5 566 579 10.1108/14635770610690410

[B49] Gencer C Gürpinar D Analytic network process in supplier selection: a case study in an electronic firm Appl Math Model 2007 31 11 2475 2486 10.1016/j.apm.2006.10.002

[B50] Saaty TL The Analytic Hierarchy Process: Planning, Priority Setting, Resource Allocation 1980 New York: McGraw-Hill International Book Co

[B51] Saaty TL Takizawz M Dependence and independence: from linear hierarchies to nonlinear networks Eur J Oper Res 1986 26 2 229 237 10.1016/0377-2217(86)90184-0

[B52] Saaty TL Decision Making with Dependence and Feedback: The Analytic Network Process 1996 Pittsburgh, PA: RWS Publications

[B53] Leung LC Cao D On the efficacy of modeling multi-attribute decision problems using AHP and Sinarchy Eur J Oper Res 2001 132 1 39 49 10.1016/S0377-2217(00)00111-9

[B54] Meade L Sarkis J Strategic analysis of logistics and supply chain management systems using the analytical network process Transp Res Part E: Logist Trans Rev 1998 34 3 201 215 10.1016/S1366-5545(98)00012-X

[B55] Jharkharia S Shankar R Selection of logistics service provider: an analytic network process (ANP) approach Omega 2007 35 3 274 289 10.1016/j.omega.2005.06.005

[B56] Saaty TL Theory and Applications of the Analytic Network Process: Decision Making with Benefits, Opportunities, Costs, and Risks 2005 Pittsburgh, PA: RWS Publications

